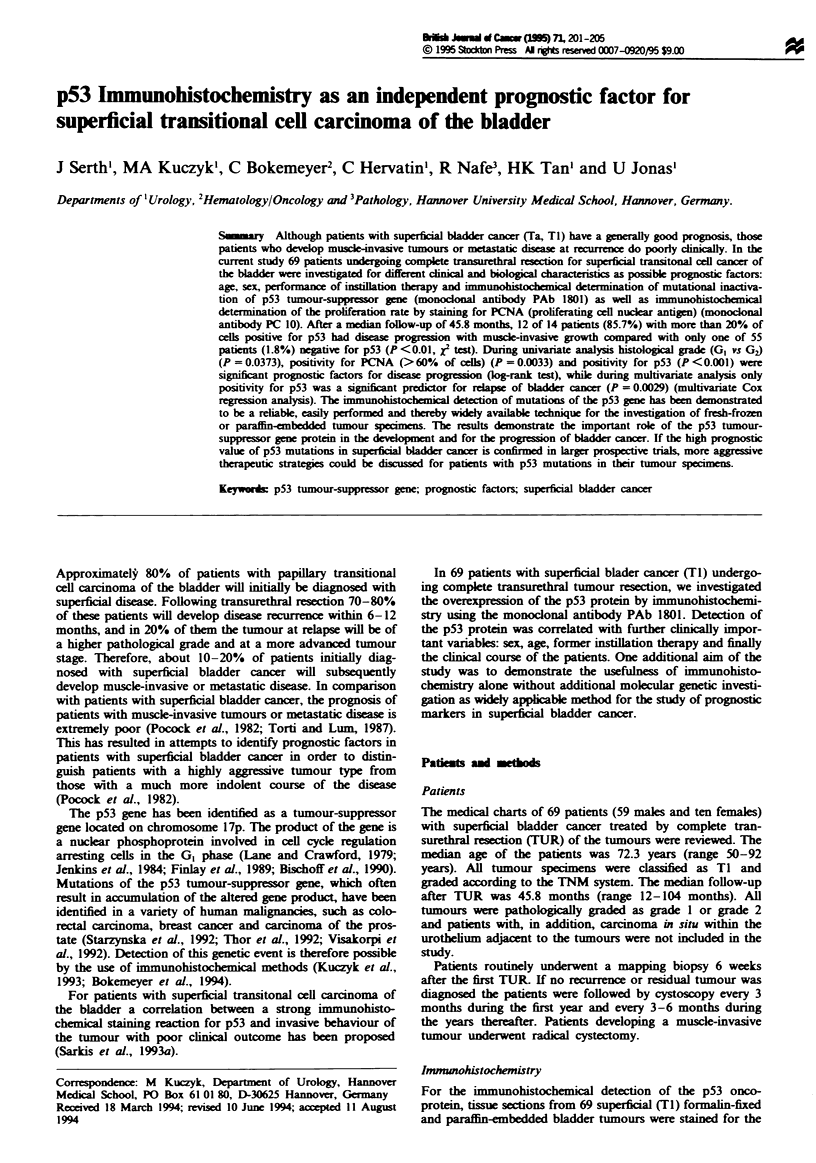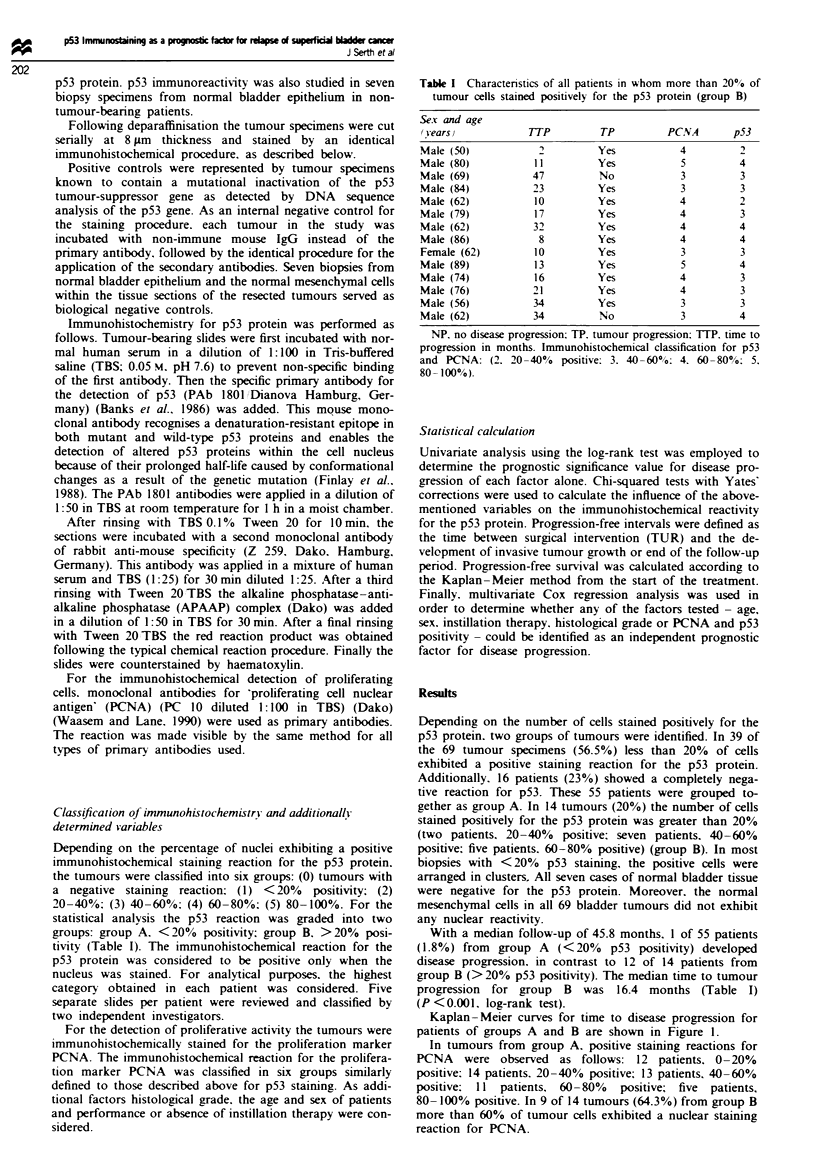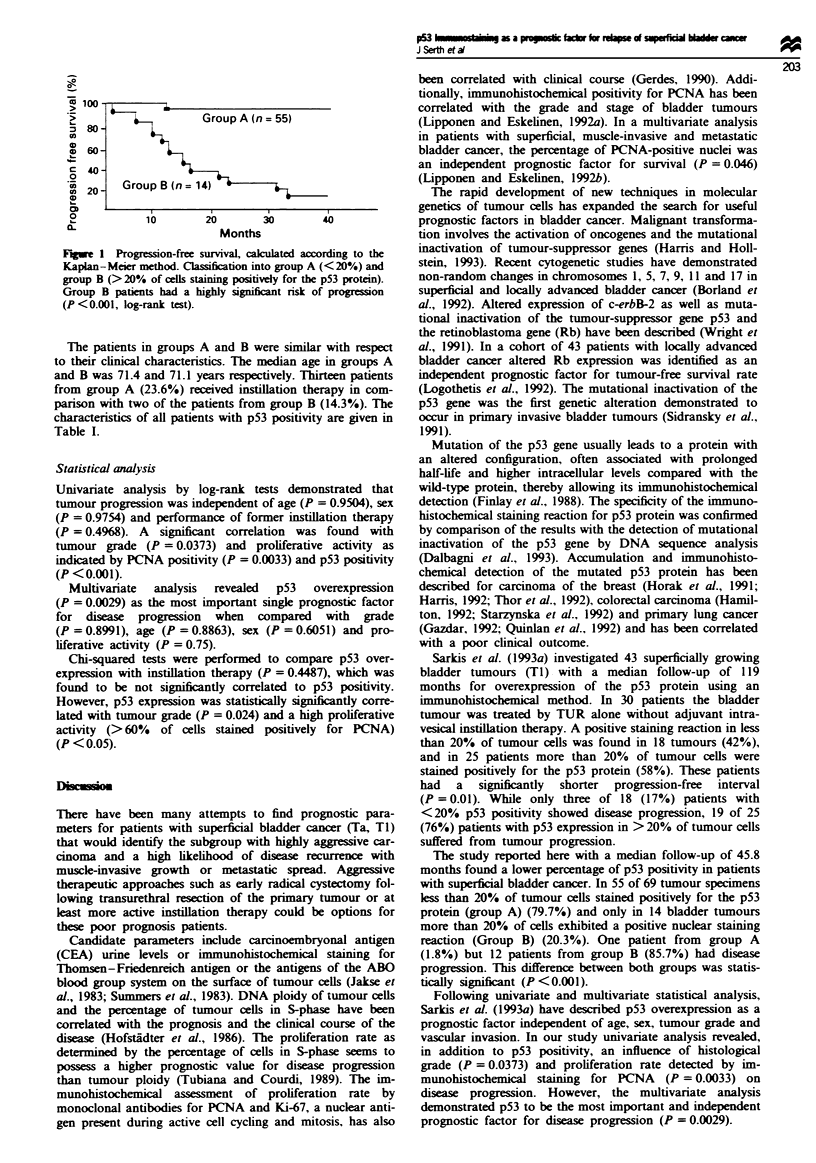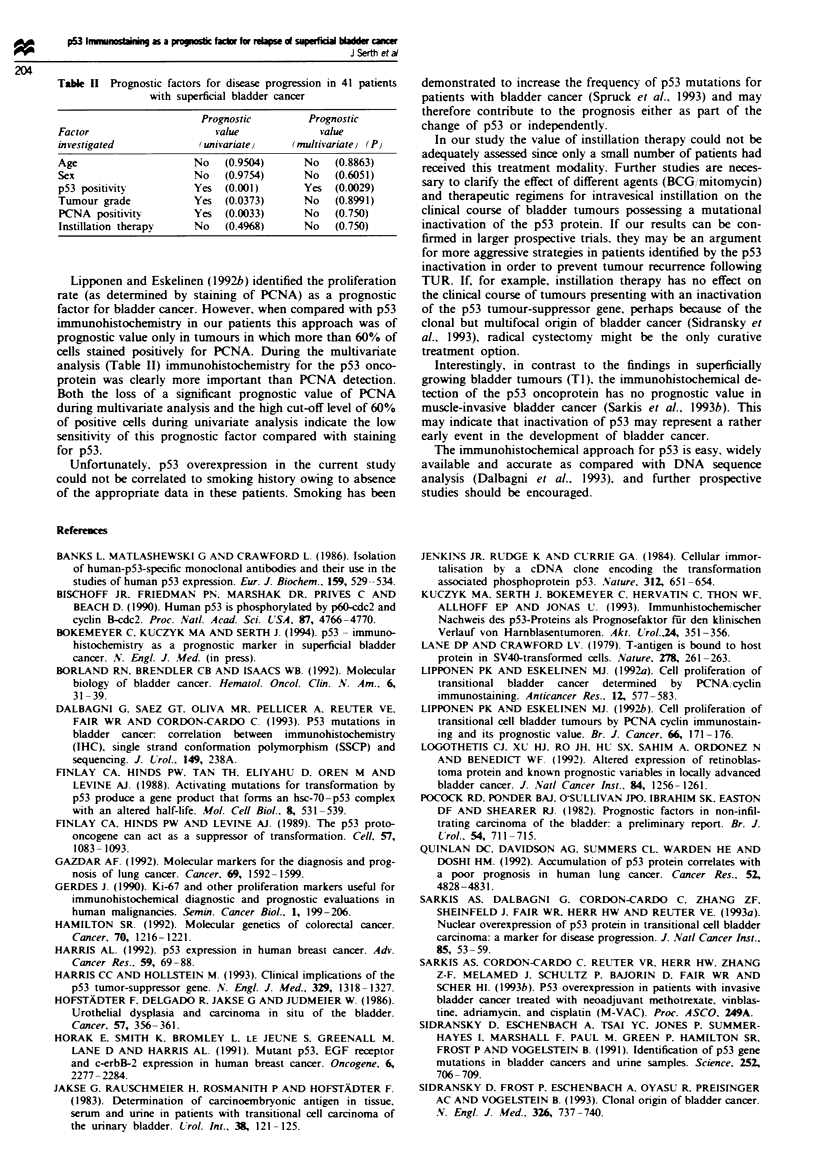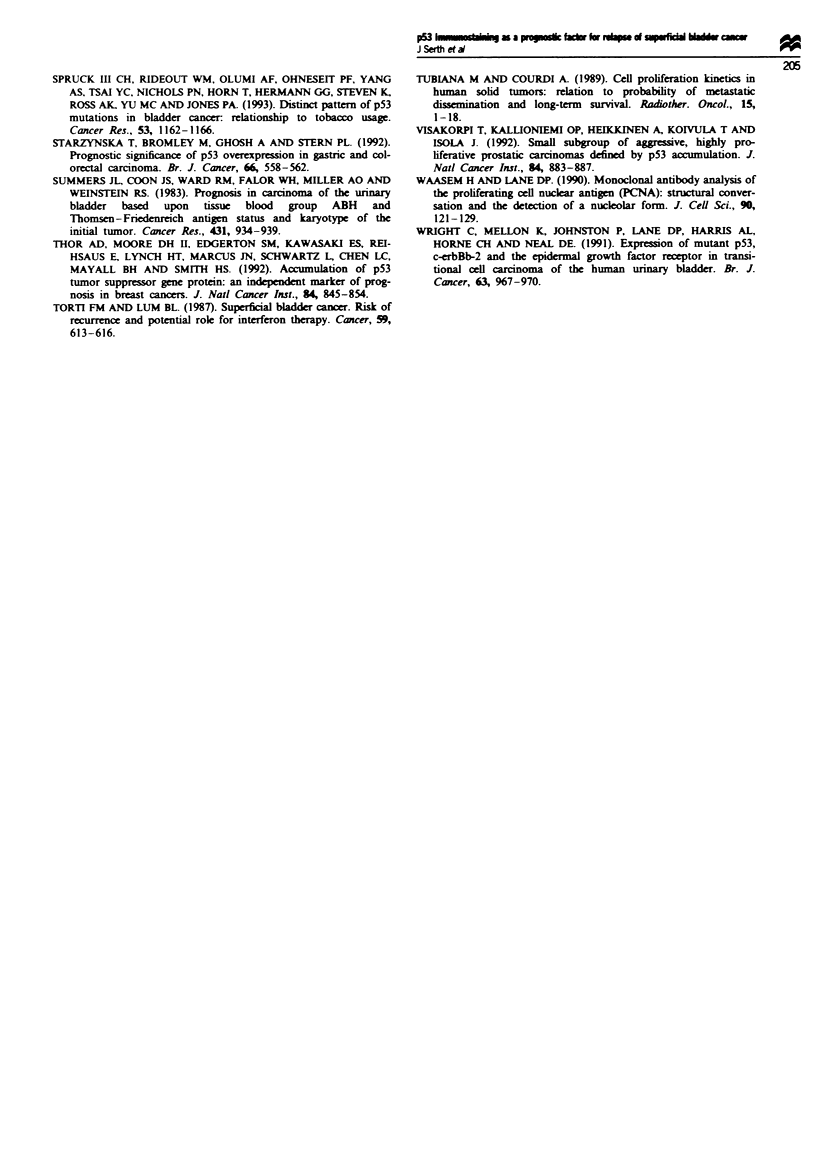# p53 immunohistochemistry as an independent prognostic factor for superficial transitional cell carcinoma of the bladder.

**DOI:** 10.1038/bjc.1995.41

**Published:** 1995-01

**Authors:** J. Serth, M. A. Kuczyk, C. Bokemeyer, C. Hervatin, R. Nafe, H. K. Tan, U. Jonas

**Affiliations:** Department of Urology, Hannover University Medical School, Germany.

## Abstract

Although patients with superficial bladder cancer (Ta, T1) have a generally good prognosis, those patients who develop muscle-invasive tumours or metastatic disease at recurrence do poorly clinically. In the current study 69 patients undergoing complete transurethral resection for superficial transitional cell cancer of the bladder were investigated for different clinical and biological characteristics as possible prognostic factors: age, sex, performance of instillation therapy and immunohistochemical determination of mutational inactivation of p53 tumour-suppressor gene (monoclonal antibody PAb 1801) as well as immunohistochemical determination of the proliferation rate by staining for PCNA (proliferating cell nuclear antigen) (monoclonal antibody PC 10). After a median follow-up of 45.8 months, 12 of 14 patients (85.7%) with more than 20% of cells positive for p53 had disease progression with muscle-invasive growth compared with only one of 55 patients (1.8%) negative for p53 (P < 0.01, chi 2 test). During univariate analysis histological grade (G1 vs G2) (P = 0.0373), positivity for PCNA (> 60% of cells) (P = 0.0033) and positivity for p53 (P < 0.001) were significant prognostic factors for disease progression (log-rank test), while during multivariate analysis only positivity for p53 was a significant predictor for relapse of bladder cancer (P = 0.0029) (multivariate Cox regression analysis). The immunohistochemical detection of mutations of the p53 gene has been demonstrated to be a reliable, easily performed and thereby widely available technique for the investigation of fresh-frozen or paraffin-embedded tumour specimens. The results demonstrate the important role of the p53 tumour-suppressor gene protein in the development and for the progression of bladder cancer. If the high prognostic value of p53 mutations in superficial bladder cancer is confirmed in larger prospective trials, more aggressive therapeutic strategies could be discussed for patients with p53 mutations in their tumour specimens.


© 1995 Stockton Press All rights reserved 0007-0920/95 $9.00

### p53 Immunohistochemistry as an independent prognostic factor for superficial transitional cell carcinoma of the bladder

J Serth[1], MA Kuczyk[1], C Bokemeyer[2], C Hervatin[1], R Nafe[3], HK Tan[1] and U Jonas[1]

*Departments of [1]Urology, [2]Hematology/Oncology and [3]Pathology, Hannover University Medical School, Hannover, Germany.*

**Summary** Although patients with superficial bladder cancer (Ta, T1) have a generally good prognosis, those patients who develop muscle-invasive tumours or metastatic disease at recurrence do poorly clinically. In the current study 69 patients undergoing complete transurethral resection for superficial transitonal cell cancer of the bladder were investigated for different clinical and biological characteristics as possible prognostic factors: age, sex, performance of instillation therapy and immunohistochemical determination of mutational inactivation of p53 tumour-suppressor gene (monoclonal antibody PAb 1801) as well as immunohistochemical determination of the proliferation rate by staining for PCNA (proliferating cell nuclear antigen) (monoclonal antibody PC 10). After a median follow-up of 45.8 months, 12 of 14 patients (85.7%) with more than 20% of cells positive for p53 had disease progression with muscle-invasive growth compared with only one of 55 patients (1.8%) negative for p53 ($P < 0.01$, $\chi^2$ test). During univariate analysis histological grade ($G_1$ *vs* $G_2$) ($P = 0.0373$), positivity for PCNA (>60% of cells) ($P = 0.0033$) and positivity for p53 ($P < 0.001$) were significant prognostic factors for disease progression (log-rank test), while during multivariate analysis only positivity for p53 was a significant predictor for relapse of bladder cancer ($P = 0.0029$) (multivariate Cox regression analysis). The immunohistochemical detection of mutations of the p53 gene has been demonstrated to be a reliable, easily performed and thereby widely available technique for the investigation of fresh-frozen or paraffin-embedded tumour specimens. The results demonstrate the important role of the p53 tumour-suppressor gene protein in the development and for the progression of bladder cancer. If the high prognostic value of p53 mutations in superficial bladder cancer is confirmed in larger prospective trials, more aggressive therapeutic strategies could be discussed for patients with p53 mutations in their tumour specimens.

**Keywords:** p53 tumour-suppressor gene; prognostic factors; superficial bladder cancer

Approximately 80% of patients with papillary transitional cell carcinoma of the bladder will initially be diagnosed with superficial disease. Following transurethral resection 70-80% of these patients will develop disease recurrence within 6-12 months, and in 20% of them the tumour at relapse will be of a higher pathological grade and at a more advanced tumour stage. Therefore, about 10-20% of patients initially diagnosed with superficial bladder cancer will subsequently develop muscle-invasive or metastatic disease. In comparison with patients with superficial bladder cancer, the prognosis of patients with muscle-invasive tumours or metastatic disease is extremely poor (Pocock *et al.*, 1982; Torti and Lum, 1987). This has resulted in attempts to identify prognostic factors in patients with superficial bladder cancer in order to distinguish patients with a highly aggressive tumour type from those with a much more indolent course of the disease (Pocock *et al.*, 1982).

The p53 gene has been identified as a tumour-suppressor gene located on chromosome 17p. The product of the gene is a nuclear phosphoprotein involved in cell cycle regulation arresting cells in the $G_1$ phase (Lane and Crawford, 1979; Jenkins *et al.*, 1984; Finlay *et al.*, 1989; Bischoff *et al.*, 1990). Mutations of the p53 tumour-suppressor gene, which often result in accumulation of the altered gene product, have been identified in a variety of human malignancies, such as colorectal carcinoma, breast cancer and carcinoma of the prostate (Starzynska *et al.*, 1992; Thor *et al.*, 1992; Visakorpi *et al.*, 1992). Detection of this genetic event is therefore possible by the use of immunohistochemical methods (Kuczyk *et al.*, 1993; Bokemeyer *et al.*, 1994).

For patients with superficial transitonal cell carcinoma of the bladder a correlation between a strong immunohistochemical staining reaction for p53 and invasive behaviour of the tumour with poor clinical outcome has been proposed (Sarkis *et al.*, 1993*a*).

Correspondence: M Kuczyk, Department of Urology, Hannover Medical School, PO Box 61 01 80, D-30625 Hannover, Germany
Received 18 March 1994; revised 10 June 1994; accepted 11 August 1994

In 69 patients with superficial blader cancer (T1) undergoing complete transurethral tumour resection, we investigated the overexpression of the p53 protein by immunohistochemistry using the monoclonal antibody PAb 1801. Detection of the p53 protein was correlated with further clinically important variables: sex, age, former instillation therapy and finally the clinical course of the patients. One additional aim of the study was to demonstrate the usefulness of immunohistochemistry alone without additional molecular genetic investigation as widely applicable method for the study of prognostic markers in superficial bladder cancer.

### Patients and methods

### *Patients*

The medical charts of 69 patients (59 males and ten females) with superficial bladder cancer treated by complete transurethral resection (TUR) of the tumours were reviewed. The median age of the patients was 72.3 years (range 50-92 years). All tumour specimens were classified as T1 and graded according to the TNM system. The median follow-up after TUR was 45.8 months (range 12-104 months). All tumours were pathologically graded as grade 1 or grade 2 and patients with, in addition, carcinoma *in situ* within the urothelium adjacent to the tumours were not included in the study.

Patients routinely underwent a mapping biopsy 6 weeks after the first TUR. If no recurrence or residual tumour was diagnosed the patients were followed by cystoscopy every 3 months during the first year and every 3-6 months during the years thereafter. Patients developing a muscle-invasive tumour underwent radical cystectomy.

### *Immunohistochemistry*

For the immunohistochemical detection of the p53 oncoprotein, tissue sections from 69 superficial (T1) formalin-fixed and paraffin-embedded bladder tumours were stained for the p53 protein. p53 immunoreactivity was also studied in seven biopsy specimens from normal bladder epithelium in non-tumour-bearing patients.

Following deparaffinisation the tumour specimens were cut serially at 8 µm thickness and stained by an identical immunohistochemical procedure, as described below.

Positive controls were represented by tumour specimens known to contain a mutational inactivation of the p53 tumour-suppressor gene as detected by DNA sequence analysis of the p53 gene. As an internal negative control for the staining procedure, each tumour in the study was incubated with non-immune mouse IgG instead of the primary antibody, followed by the identical procedure for the application of the secondary antibodies. Seven biopsies from normal bladder epithelium and the normal mesenchymal cells within the tissue sections of the resected tumours served as biological negative controls.

Immunohistochemistry for p53 protein was performed as follows. Tumour-bearing slides were first incubated with normal human serum in a dilution of 1:100 in Tris-buffered saline (TBS; 0.05 M, pH 7.6) to prevent non-specific binding of the first antibody. Then the specific primary antibody for the detection of p53 (PAb 1801/Dianova Hamburg, Germany) (Banks *et al.*, 1986) was added. This mouse monoclonal antibody recognises a denaturation-resistant epitope in both mutant and wild-type p53 proteins and enables the detection of altered p53 proteins within the cell nucleus because of their prolonged half-life caused by conformational changes as a result of the genetic mutation (Finlay *et al.*, 1988). The PAb 1801 antibodies were applied in a dilution of 1:50 in TBS at room temperature for 1 h in a moist chamber.

After rinsing with TBS/0.1% Tween 20 for 10 min, the sections were incubated with a second monoclonal antibody of rabbit anti-mouse specificity (Z 259, Dako, Hamburg, Germany). This antibody was applied in a mixture of human serum and TBS (1:25) for 30 min diluted 1:25. After a third rinsing with Tween 20/TBS the alkaline phosphatase-anti-alkaline phosphatase (APAAP) complex (Dako) was added in a dilution of 1:50 in TBS for 30 min. After a final rinsing with Tween 20/TBS the red reaction product was obtained following the typical chemical reaction procedure. Finally the slides were counterstained by haematoxylin.

For the immunohistochemical detection of proliferating cells, monoclonal antibodies for 'proliferating cell nuclear antigen' (PCNA) (PC 10 diluted 1:100 in TBS) (Dako) (Waasem and Lane, 1990) were used as primary antibodies. The reaction was made visible by the same method for all types of primary antibodies used.

*Classification of immunohistochemistry and additionally determined variables*

Depending on the percentage of nuclei exhibiting a positive immunohistochemical staining reaction for the p53 protein, the tumours were classified into six groups: (0) tumours with a negative staining reaction; (1) <20% positivity; (2) 20–40%; (3) 40–60%; (4) 60–80%; (5) 80–100%. For the statistical analysis the p53 reaction was graded into two groups: group A, <20% positivity; group B, >20% positivity (Table I). The immunohistochemical reaction for the p53 protein was considered to be positive only when the nucleus was stained. For analytical purposes, the highest category obtained in each patient was considered. Five separate slides per patient were reviewed and classified by two independent investigators.

For the detection of proliferative activity the tumours were immunohistochemically stained for the proliferation marker PCNA. The immunohistochemical reaction for the proliferation marker PCNA was classified in six groups similarly defined to those described above for p53 staining. As additional factors histological grade, the age and sex of patients and performance or absence of instillation therapy were considered.

**Table I** Characteristics of all patients in whom more than 20% of tumour cells stained positively for the p53 protein (group B)

| *Sex and age (years)* | *TTP* | *TP* | *PCNA* | *p53* |
|---|---|---|---|---|
| Male (50) | 2 | Yes | 4 | 2 |
| Male (80) | 11 | Yes | 5 | 4 |
| Male (69) | 47 | No | 3 | 3 |
| Male (84) | 23 | Yes | 3 | 3 |
| Male (62) | 10 | Yes | 4 | 2 |
| Male (79) | 17 | Yes | 4 | 3 |
| Male (62) | 32 | Yes | 4 | 4 |
| Male (86) | 8 | Yes | 4 | 4 |
| Female (62) | 10 | Yes | 3 | 3 |
| Male (89) | 13 | Yes | 5 | 4 |
| Male (74) | 16 | Yes | 4 | 3 |
| Male (76) | 21 | Yes | 4 | 3 |
| Male (56) | 34 | Yes | 3 | 3 |
| Male (62) | 34 | No | 3 | 4 |

NP, no disease progression; TP, tumour progression; TTP, time to progression in months. Immunohistochemical classification for p53 and PCNA: (2, 20–40% positive; 3, 40–60%; 4, 60–80%; 5, 80–100%).

*Statistical calculation*

Univariate analysis using the log-rank test was employed to determine the prognostic significance value for disease progression of each factor alone. Chi-squared tests with Yates' corrections were used to calculate the influence of the above-mentioned variables on the immunohistochemical reactivity for the p53 protein. Progression-free intervals were defined as the time between surgical intervention (TUR) and the development of invasive tumour growth or end of the follow-up period. Progression-free survival was calculated according to the Kaplan–Meier method from the start of the treatment. Finally, multivariate Cox regression analysis was used in order to determine whether any of the factors tested – age, sex, instillation therapy, histological grade or PCNA and p53 positivity – could be identified as an independent prognostic factor for disease progression.

### Results

Depending on the number of cells stained positively for the p53 protein, two groups of tumours were identified. In 39 of the 69 tumour specimens (56.5%) less than 20% of cells exhibited a positive staining reaction for the p53 protein. Additionally, 16 patients (23%) showed a completely negative reaction for p53. These 55 patients were grouped together as group A. In 14 tumours (20%) the number of cells stained positively for the p53 protein was greater than 20% (two patients, 20–40% positive; seven patients, 40–60% positive; five patients, 60–80% positive) (group B). In most biopsies with <20% p53 staining, the positive cells were arranged in clusters. All seven cases of normal bladder tissue were negative for the p53 protein. Moreover, the normal mesenchymal cells in all 69 bladder tumours did not exhibit any nuclear reactivity.

With a median follow-up of 45.8 months, 1 of 55 patients (1.8%) from group A (<20% p53 positivity) developed disease progression, in contrast to 12 of 14 patients from group B (>20% p53 positivity). The median time to tumour progression for group B was 16.4 months (Table I) ($P < 0.001$, log-rank test).

Kaplan–Meier curves for time to disease progression for patients of groups A and B are shown in Figure 1.

In tumours from group A, positive staining reactions for PCNA were observed as follows: 12 patients, 0–20% positive; 14 patients, 20–40% positive; 13 patients, 40–60% positive; 11 patients, 60–80% positive; five patients, 80–100% positive. In 9 of 14 tumours (64.3%) from group B more than 60% of tumour cells exhibited a nuclear staining reaction for PCNA.

**Figure 1** Progression-free survival, calculated according to the Kaplan-Meier method. Classification into group A (<20%) and group B (>20% of cells staining positively for the p53 protein). Group B patients had a highly significant risk of progression ($P<0.001$, log-rank test).

The patients in groups A and B were similar with respect to their clinical characteristics. The median age in groups A and B was 71.4 and 71.1 years respectively. Thirteen patients from group A (23.6%) received instillation therapy in comparison with two of the patients from group B (14.3%). The characteristics of all patients with p53 positivity are given in Table I.

### *Statistical analysis*

Univariate analysis by log-rank tests demonstrated that tumour progression was independent of age ($P = 0.9504$), sex ($P = 0.9754$) and performance of former instillation therapy ($P = 0.4968$). A significant correlation was found with tumour grade ($P = 0.0373$) and proliferative activity as indicated by PCNA positivity ($P = 0.0033$) and p53 positivity ($P<0.001$).

Multivariate analysis revealed p53 overexpression ($P = 0.0029$) as the most important single prognostic factor for disease progression when compared with grade ($P = 0.8991$), age ($P = 0.8863$), sex ($P = 0.6051$) and proliferative activity ($P = 0.75$).

Chi-squared tests were performed to compare p53 overexpression with instillation therapy ($P = 0.4487$), which was found to be not significantly correlated to p53 positivity. However, p53 expression was statistically significantly correlated with tumour grade ($P = 0.024$) and a high proliferative activity (>60% of cells stained positively for PCNA) ($P<0.05$).

### Discussion

There have been many attempts to find prognostic parameters for patients with superficial bladder cancer (Ta, T1) that would identify the subgroup with highly aggressive carcinoma and a high likelihood of disease recurrence with muscle-invasive growth or metastatic spread. Aggressive therapeutic approaches such as early radical cystectomy following transurethral resection of the primary tumour or at least more active instillation therapy could be options for these poor prognosis patients.

Candidate parameters include carcinoembryonal antigen (CEA) urine levels or immunohistochemical staining for Thomsen-Friedenreich antigen or the antigens of the ABO blood group system on the surface of tumour cells (Jakse *et al.*, 1983; Summers *et al.*, 1983). DNA ploidy of tumour cells and the percentage of tumour cells in S-phase have been correlated with the prognosis and the clinical course of the disease (Hofstädter *et al.*, 1986). The proliferation rate as determined by the percentage of cells in S-phase seems to possess a higher prognostic value for disease progression than tumour ploidy (Tubiana and Courdi, 1989). The immunohistochemical assessment of proliferation rate by monoclonal antibodies for PCNA and Ki-67, a nuclear antigen present during active cell cycling and mitosis, has also been correlated with clinical course (Gerdes, 1990). Additionally, immunohistochemical positivity for PCNA has been correlated with the grade and stage of bladder tumours (Lipponen and Eskelinen, 1992*a*). In a multivariate analysis in patients with superficial, muscle-invasive and metastatic bladder cancer, the percentage of PCNA-positive nuclei was an independent prognostic factor for survival ($P = 0.046$) (Lipponen and Eskelinen, 1992*b*).

The rapid development of new techniques in molecular genetics of tumour cells has expanded the search for useful prognostic factors in bladder cancer. Malignant transformation involves the activation of oncogenes and the mutational inactivation of tumour-suppressor genes (Harris and Hollstein, 1993). Recent cytogenetic studies have demonstrated non-random changes in chromosomes 1, 5, 7, 9, 11 and 17 in superficial and locally advanced bladder cancer (Borland *et al.*, 1992). Altered expression of c-*erb*B-2 as well as mutational inactivation of the tumour-suppressor gene p53 and the retinoblastoma gene (Rb) have been described (Wright *et al.*, 1991). In a cohort of 43 patients with locally advanced bladder cancer altered Rb expression was identified as an independent prognostic factor for tumour-free survival rate (Logothetis *et al.*, 1992). The mutational inactivation of the p53 gene was the first genetic alteration demonstrated to occur in primary invasive bladder tumours (Sidransky *et al.*, 1991).

Mutation of the p53 gene usually leads to a protein with an altered configuration, often associated with prolonged half-life and higher intracellular levels compared with the wild-type protein, thereby allowing its immunohistochemical detection (Finlay *et al.*, 1988). The specificity of the immunohistochemical staining reaction for p53 protein was confirmed by comparison of the results with the detection of mutational inactivation of the p53 gene by DNA sequence analysis (Dalbagni *et al.*, 1993). Accumulation and immunohistochemical detection of the mutated p53 protein has been described for carcinoma of the breast (Horak *et al.*, 1991; Harris, 1992; Thor *et al.*, 1992), colorectal carcinoma (Hamilton, 1992; Starzynska *et al.*, 1992) and primary lung cancer (Gazdar, 1992; Quinlan *et al.*, 1992) and has been correlated with a poor clinical outcome.

Sarkis *et al.* (1993*a*) investigated 43 superficially growing bladder tumours (T1) with a median follow-up of 119 months for overexpression of the p53 protein using an immunohistochemical method. In 30 patients the bladder tumour was treated by TUR alone without adjuvant intravesical instillation therapy. A positive staining reaction in less than 20% of tumour cells was found in 18 tumours (42%), and in 25 patients more than 20% of tumour cells were stained positively for the p53 protein (58%). These patients had a significantly shorter progression-free interval ($P = 0.01$). While only three of 18 (17%) patients with <20% p53 positivity showed disease progression, 19 of 25 (76%) patients with p53 expression in >20% of tumour cells suffered from tumour progression.

The study reported here with a median follow-up of 45.8 months found a lower percentage of p53 positivity in patients with superficial bladder cancer. In 55 of 69 tumour specimens less than 20% of tumour cells stained positively for the p53 protein (group A) (79.7%) and only in 14 bladder tumours more than 20% of cells exhibited a positive nuclear staining reaction (Group B) (20.3%). One patient from group A (1.8%) but 12 patients from group B (85.7%) had disease progression. This difference between both groups was statistically significant ($P<0.001$).

Following univariate and multivariate statistical analysis, Sarkis *et al.* (1993*a*) have described p53 overexpression as a prognostic factor independent of age, sex, tumour grade and vascular invasion. In our study univariate analysis revealed, in addition to p53 positivity, an influence of histological grade ($P = 0.0373$) and proliferation rate detected by immunohistochemical staining for PCNA ($P = 0.0033$) on disease progression. However, the multivariate analysis demonstrated p53 to be the most important and independent prognostic factor for disease progression ($P = 0.0029$).

**Table II** Prognostic factors for disease progression in 41 patients with superficial bladder cancer

| *Factor investigated* | *Prognostic value (univariate)* | *Prognostic value (multivariate) (P)* |
|---|---|---|
| Age | No (0.9504) | No (0.8863) |
| Sex | No (0.9754) | No (0.6051) |
| p53 positivity | Yes (0.001) | Yes (0.0029) |
| Tumour grade | Yes (0.0373) | No (0.8991) |
| PCNA positivity | Yes (0.0033) | No (0.750) |
| Instillation therapy | No (0.4968) | No (0.750) |

Lipponen and Eskelinen (1992*b*) identified the proliferation rate (as determined by staining of PCNA) as a prognostic factor for bladder cancer. However, when compared with p53 immunohistochemistry in our patients this approach was of prognostic value only in tumours in which more than 60% of cells stained positively for PCNA. During the multivariate analysis (Table II) immunohistochemistry for the p53 oncoprotein was clearly more important than PCNA detection. Both the loss of a significant prognostic value of PCNA during multivariate analysis and the high cut-off level of 60% of positive cells during univariate analysis indicate the low sensitivity of this prognostic factor compared with staining for p53.

Unfortunately, p53 overexpression in the current study could not be correlated to smoking history owing to absence of the appropriate data in these patients. Smoking has been demonstrated to increase the frequency of p53 mutations for patients with bladder cancer (Spruck *et al.*, 1993) and may therefore contribute to the prognosis either as part of the change of p53 or independently.

In our study the value of instillation therapy could not be adequately assessed since only a small number of patients had received this treatment modality. Further studies are necessary to clarify the effect of different agents (BCG/mitomycin) and therapeutic regimens for intravesical instillation on the clinical course of bladder tumours possessing a mutational inactivation of the p53 protein. If our results can be confirmed in larger prospective trials, they may be an argument for more aggressive strategies in patients identified by the p53 inactivation in order to prevent tumour recurrence following TUR. If, for example, instillation therapy has no effect on the clinical course of tumours presenting with an inactivation of the p53 tumour-suppressor gene, perhaps because of the clonal but multifocal origin of bladder cancer (Sidransky *et al.*, 1993), radical cystectomy might be the only curative treatment option.

Interestingly, in contrast to the findings in superficially growing bladder tumours (T1), the immunohistochemical detection of the p53 oncoprotein has no prognostic value in muscle-invasive bladder cancer (Sarkis *et al.*, 1993*b*). This may indicate that inactivation of p53 may represent a rather early event in the development of bladder cancer.

The immunohistochemical approach for p53 is easy, widely available and accurate as compared with DNA sequence analysis (Dalbagni *et al.*, 1993), and further prospective studies should be encouraged.

### References

BANKS L, MATLASHEWSKI G AND CRAWFORD L. (1986). Isolation of human-p53-specific monoclonal antibodies and their use in the studies of human p53 expression. *Eur. J. Biochem.*, **159**, 529–534.

BISCHOFF JR, FRIEDMAN PN, MARSHAK DR, PRIVES C AND BEACH D. (1990). Human p53 is phosphorylated by p60-cdc2 and cyclin B-cdc2. *Proc. Natl. Acad. Sci. USA*, **87**, 4766–4770.

BOKEMEYER C, KUCZYK MA AND SERTH J. (1994). p53 – immunohistochemistry as a prognostic marker in superficial bladder cancer. *N. Engl. J. Med.* (in press).

BORLAND RN, BRENDLER CB AND ISAACS WB. (1992). Molecular biology of bladder cancer. *Hematol. Oncol. Clin. N. Am.*, **6**, 31–39.

DALBAGNI G, SAEZ GT, OLIVA MR, PELLICER A, REUTER VE, FAIR WR AND CORDON-CARDO C. (1993). P53 mutations in bladder cancer: correlation between immunohistochemistry (IHC), single strand conformation polymorphism (SSCP) and sequencing. *J. Urol.*, **149**, 238A.

FINLAY CA, HINDS PW, TAN TH, ELIYAHU D, OREN M AND LEVINE AJ. (1988). Activating mutations for transformation by p53 produce a gene product that forms an hsc-70–p53 complex with an altered half-life. *Mol. Cell Biol.*, **8**, 531–539.

FINLAY CA, HINDS PW AND LEVINE AJ. (1989). The p53 proto-oncogene can act as a suppressor of transformation. *Cell*, **57**, 1083–1093.

GAZDAR AF. (1992). Molecular markers for the diagnosis and prognosis of lung cancer. *Cancer*, **69**, 1592–1599.

GERDES J. (1990). Ki-67 and other proliferation markers useful for immunohistochemical diagnostic and prognostic evaluations in human malignancies. *Semin. Cancer Biol.*, **1**, 199–206.

HAMILTON SR. (1992). Molecular genetics of colorectal cancer. *Cancer*, **70**, 1216–1221.

HARRIS AL. (1992). p53 expression in human breast cancer. *Adv. Cancer Res.*, **59**, 69–88.

HARRIS CC AND HOLLSTEIN M. (1993). Clinical implications of the p53 tumor-suppressor gene. *N. Engl. J. Med.*, **329**, 1318–1327.

HOFSTÄDTER F, DELGADO R, JAKSE G AND JUDMEIER W. (1986). Urothelial dysplasia and carcinoma in situ of the bladder. *Cancer*, **57**, 356–361.

HORAK E, SMITH K, BROMLEY L, LE JEUNE S, GREENALL M, LANE D AND HARRIS AL. (1991). Mutant p53, EGF receptor and c-erbB-2 expression in human breast cancer. *Oncogene*, **6**, 2277–2284.

JAKSE G, RAUSCHMEIER H, ROSMANITH P AND HOFSTÄDTER F. (1983). Determination of carcinoembryonic antigen in tissue, serum and urine in patients with transitional cell carcinoma of the urinary bladder. *Urol. Int.*, **38**, 121–125.

JENKINS JR, RUDGE K AND CURRIE GA. (1984). Cellular immortalisation by a cDNA clone encoding the transformation associated phosphoprotein p53. *Nature*, **312**, 651–654.

KUCZYK MA, SERTH J, BOKEMEYER C, HERVATIN C, THON WF, ALLHOFF EP AND JONAS U. (1993). Immunhistochemischer Nachweis des p53-Proteins als Prognosefaktor für den klinischen Verlauf von Harnblasentumoren. *Akt. Urol.*, **24**, 351–356.

LANE DP AND CRAWFORD LV. (1979). T-antigen is bound to host protein in SV40-transformed cells. *Nature*, **278**, 261–263.

LIPPONEN PK AND ESKELINEN MJ. (1992*a*). Cell proliferation of transitional bladder cancer determined by PCNA/cyclin immunostaining. *Anticancer Res.*, **12**, 577–583.

LIPPONEN PK AND ESKELINEN MJ. (1992*b*). Cell proliferation of transitional cell bladder tumours by PCNA cyclin immunostaining and its prognostic value. *Br. J. Cancer*, **66**, 171–176.

LOGOTHETIS CJ, XU HJ, RO JH, HU SX, SAHIM A, ORDONEZ N AND BENEDICT WF. (1992). Altered expression of retinoblastoma protein and known prognostic variables in locally advanced bladder cancer. *J. Natl Cancer Inst.*, **84**, 1256–1261.

POCOCK RD, PONDER BAJ, O'SULLIVAN JPO, IBRAHIM SK, EASTON DF AND SHEARER RJ. (1982). Prognostic factors in non-infiltrating carcinoma of the bladder: a preliminary report. *Br. J. Urol.*, **54**, 711–715.

QUINLAN DC, DAVIDSON AG, SUMMERS CL, WARDEN HE AND DOSHI HM. (1992). Accumulation of p53 protein correlates with a poor prognosis in human lung cancer. *Cancer Res.*, **52**, 4828–4831.

SARKIS AS, DALBAGNI G, CORDON-CARDO C, ZHANG ZF, SHEINFELD J, FAIR WR, HERR HW AND REUTER VE. (1993*a*). Nuclear overexpression of p53 protein in transitional cell bladder carcinoma: a marker for disease progression. *J. Natl Cancer Inst.*, **85**, 53–59.

SARKIS AS, CORDON-CARDO C, REUTER VR, HERR HW, ZHANG Z-F, MELAMED J, SCHULTZ P, BAJORIN D, FAIR WR AND SCHER HI. (1993*b*). P53 overexpression in patients with invasive bladder cancer treated with neoadjuvant methotrexate, vinblastine, adriamycin, and cisplatin (M-VAC). *Proc. ASCO*, **249A**.

SIDRANSKY D, ESCHENBACH A, TSAI YC, JONES P, SUMMERHAYES I, MARSHALL F, PAUL M, GREEN P, HAMILTON SR, FROST P AND VOGELSTEIN B. (1991). Identification of p53 gene mutations in bladder cancers and urine samples. *Science*, **252**, 706–709.

SIDRANSKY D, FROST P, ESCHENBACH A, OYASU R, PREISINGER AC AND VOGELSTEIN B. (1993). Clonal origin of bladder cancer. *N. Engl. J. Med.*, **326**, 737–740.

SPRUCK III CH, RIDEOUT WM, OLUMI AF, OHNESEIT PF, YANG AS, TSAI YC, NICHOLS PN, HORN T, HERMANN GG, STEVEN K, ROSS AK, YU MC AND JONES PA. (1993). Distinct pattern of p53 mutations in bladder cancer: relationship to tobacco usage. *Cancer Res.*, **53**, 1162–1166.

STARZYNSKA T, BROMLEY M, GHOSH A AND STERN PL. (1992). Prognostic significance of p53 overexpression in gastric and colorectal carcinoma. *Br. J. Cancer*, **66**, 558–562.

SUMMERS JL, COON JS, WARD RM, FALOR WH, MILLER AO AND WEINSTEIN RS. (1983). Prognosis in carcinoma of the urinary bladder based upon tissue blood group ABH and Thomsen-Friedenreich antigen status and karyotype of the initial tumor. *Cancer Res.*, **431**, 934–939.

THOR AD, MOORE DH II, EDGERTON SM, KAWASAKI ES, REIHSAUS E, LYNCH HT, MARCUS JN, SCHWARTZ L, CHEN LC, MAYALL BH AND SMITH HS. (1992). Accumulation of p53 tumor suppressor gene protein: an independent marker of prognosis in breast cancers. *J. Natl Cancer Inst.*, **84**, 845–854.

TORTI FM AND LUM BL. (1987). Superficial bladder cancer. Risk of recurrence and potential role for interferon therapy. *Cancer*, **59**, 613–616.

TUBIANA M AND COURDI A. (1989). Cell proliferation kinetics in human solid tumors: relation to probability of metastatic dissemination and long-term survival. *Radiother. Oncol.*, **15**, 1–18.

VISAKORPI T, KALLIONIEMI OP, HEIKKINEN A, KOIVULA T AND ISOLA J. (1992). Small subgroup of aggressive, highly proliferative prostatic carcinomas defined by p53 accumulation. *J. Natl Cancer Inst.*, **84**, 883–887.

WAASEM H AND LANE DP. (1990). Monoclonal antibody analysis of the proliferating cell nuclear antigen (PCNA): structural conversation and the detection of a nucleolar form. *J. Cell Sci.*, **90**, 121–129.

WRIGHT C, MELLON K, JOHNSTON P, LANE DP, HARRIS AL, HORNE CH AND NEAL DE. (1991). Expression of mutant p53, c-erbBb-2 and the epidermal growth factor receptor in transitional cell carcinoma of the human urinary bladder. *Br. J. Cancer*, **63**, 967–970.